# Healthcare-use for Major Infectious Disease Syndromes in an Informal Settlement in Nairobi, Kenya

**DOI:** 10.3329/jhpn.v29i2.7854

**Published:** 2011-04

**Authors:** Robert F. Breiman, Beatrice Olack, Alvin Shultz, Sanam Roder, Kabuiya Kimani, Daniel R. Feikin, Heather Burke

**Affiliations:** International Emerging Infections Program; CDC-Kenya Medical Research Institute, Nairobi, Kenya

**Keywords:** Acute respiratory infection, Diarrhoeal diseases, Febrile illness, Healthcare-seeking, Healthcare-use, Informal settlements, Pneumonia, Slums, Urbanization, Kenya

## Abstract

A healthcare-use survey was conducted in the Kibera informal settlement in Nairobi, Kenya, in July 2005 to inform subsequent surveillance in the site for infectious diseases. Sets of standardized questionnaires were administered to 1,542 caretakers and heads of households with one or more child(ren) aged less than five years. The average household-size was 5.1 (range 1-15) persons. Most (90%) resided in a single room with monthly rents of US$ 4.50-7.00. Within the previous two weeks, 49% of children (n=1,378) aged less than five years (under-five children) and 18% of persons (n=1,139) aged ≥5 years experienced febrile, diarrhoeal or respiratory illnesses. The large majority (>75%) of illnesses were associated with healthcare-seeking. While licensed clinics were the most-frequently visited settings, kiosks, unlicensed care providers, and traditional healers were also frequently visited. Expense was cited most often (50%) as the reason for not seeking healthcare. Of those who sought healthcare, 34-44% of the first and/or the only visits were made with non-licensed care providers, potentially delaying opportunities for early optimal intervention. The proportions of patients accessing healthcare facilities were higher with diarrhoeal disease and fever (but not for respiratory diseases in under-five children) than those reported from a contemporaneous study conducted in a rural area in Kenya. The findings support community-based rather than facility-based surveillance in this setting to achieve objectives for comprehensive assessment of the burden of disease.

## INTRODUCTION

During 1950-2008, the proportion of the world's population living within cities grew from 33% to 50% (1). The number of cities with >10 million people grew from 3 to 20 over the past 30 years; 15 of these ‘megacities’ were in the developing world (1). The urban population in sub-Saharan Africa is expected to triple between 2010 and 2050 to >1.2 billion people (2). This process of massive urbanization has been ongoing in many developing countries, including in sub-Saharan Africa where urbanization rates, often linked to extreme poverty (3), are the highest in the world (4). Perceived opportunities for improving the family status and for education have led many people to leave rural settings in favour of urban living environments which can be more challenging and less familiar (5). Since urban areas often cannot address the demands of an expanding population with strengthened infrastructure, the new residences are frequently seen within informal settlements, often referred to as slums, where the density of population is high, and sanitation, availability of clean water, and health services are low (5).

Due to various adverse conditions prevalent in informal settlements, transmission of pathogens that cause infectious diseases may be facilitated, resulting in life-threatening syndromes, such as severe pneumonia, diarrhoeal disease, and febrile illnesses. To identify the emerging pathogens, characterize their epidemiology and the burden of disease, and assist in identifying priorities for the prevention of diseases, the International Emerging Infections Program (IEIP) of the Centers for Disease Control and Prevention (CDC), in collaboration with the Kenya Medical Research Centre (KEMRI), established a surveillance for key infectious disease syndromes and their aetiologies. This surveillance takes place in two villages—both within Kibera, an informal settlement, representing one of the largest contiguous slums in sub-Saharan Africa (6). The area is characterized by high density of population, poor sanitary conditions, and limited access to safe water (7).

Healthcare-use patterns determine whether a clinic or hospital-based surveillance is adequate to define the incidence of diseases or whether more active approaches, such as periodic home-visits, are needed to identify a substantial proportion of illnesses. To better understand the healthcare-seeking patterns of the population under surveillance, we carried out a healthcare-use survey (HUS) within the Kibera surveillance area. Such studies can help interpret data from disease-surveillance systems, especially among people for whom delivery and quality of health services are poor (8). They provide baseline information about syndromes of substantial morbidity and mortality and can yield a method to extrapolate the burden of disease using the proportion of illnesses missed when surveillance is dependent upon attendance at a health facility. Such data improve precision in estimates of disease-incidence rates when relying on clinic-based surveillance. We set out to estimate the proportion of people with pneumonia, diarrhoea, or febrile illness symptoms, who seek care inside and outside the Kibera informal settlement and to define the places where care is sought. We also sought to characterize factors associated with specific healthcare-seeking behaviours and to identify key barriers to accessing care.

## MATERIALS AND METHODS

### Study site

The HUS and a census, used for defining the HUS study area, were conducted in July 2005 within Gatwikira and Soweto villages of Kibera (located in Nairobi, Kenya) before the population-based surveillance for infectious disease syndromes was initiated by the KEMRI/CDC in November 2005. Tabitha Clinic, managed by a collaborating partner—Carolina for Kibera—is the field/referral clinic for the surveillance project and is located in the centre of the surveillance area; the area of the surveillance villages is 0.4 sq km, with 77,000 people per sq km. The previous census (2001) by the Kenya Central Bureau of Statistics indicated that the population of these two villages was approximately 30,000.

### Data collection

#### Census

We used the census to define the catchment area for the population-based syndromic surveillance system which was in the planning stages at the time to identify the study population for the HUS and to characterize population demographics. The census consisted of house-to-house interviews of all households within Gatwikira and Soweto, as demarcated by the Kenya Central Bureau of Statistics. During 12-17 July 2005, demographers of the Kenya Central Bureau of Statistics conducted interviews, and the Bureau's cartographers drew area-maps. Heads of households were targeted for interview but when they or their spouses were not available, another adult member of the household was interviewed. When no inhabitants were present at a household structure, a neighbour was asked to provide information about whether or not the structure was occupied, and if occupied, information about the household to the best of his/her ability; at least one attempt was made to verify information provided by neighbours by re-visiting previously-empty households.

### Healthcare-use survey

Our primary focus was on healthcare-use practices for children. Thus, using the 2005 census results, all households with children aged less than five years (under-five children) residing in Gatwikira and Soweto villages were selected for participation in the HUS. While we collected data on healthcare-use practices for older children and adults, we recognized that the findings for adults may not apply to all adults in the community, i.e. adults without young children may have different practices. The KEMRI/CDC teams gathered data over a two-week period from 4 to 17 October 2005. The data-collection phase followed a one-week training of 19 community interviewers. Interviewers were grouped into teams of four or five, each supervised by a team leader. A detailed household demographics form was administered to all consenting household caretakers, followed by a form identifying household members and screening for illnesses. The duration of the survey varied from 15 to 60 minutes per household, depending upon the number of people residing in the household and the number of recent illnesses. For the screening process, the following case definitions were used:

- Fever—An illness associated with feeling hot or feverish during the two weeks before interview.
- Diarrhoea—Three or more loose stools over at least a 24-hour period during the two weeks before interview.
- Acute respiratory illness (ARI)—Cough and difficult breathing from the chest (not the nose) for more than five days and less than 30 days with at least a portion of the illness occurring during the two weeks before interview. We chose five days as the minimum duration of illness to attempt to focus on significant respiratory illness, based on a non-validated assumption that more transient illnesses (lasting <5 days) were less likely to represent serious disease.
- Pneumonia (past year)—The household member was told by a healthcare worker (such as a nurse, a doctor, or a clinical officer) that they (or their children) had ‘pneumonia’ during the year before interview.

A detailed questionnaire was administered in *Kiswahili* to participating caretakers and heads of households of all under-five children, meeting any of the illness categories. Four sets of detailed questionnaires were available for use relating to each of the four illness categories. One child aged 5-17 years and one adult aged 18 years and above were also chosen from a household if it was determined by the interviewer that the child and/or the adult had one of the illness syndromes in question within the relevant time period. The same questions concerning healthcare-seeking were asked for each syndrome. A maximum of two detailed illness-specific questionnaires was administered for any one individual. When multiple instances of diarrhoea during the previous two weeks had occurred, the questionnaire only asked about the most recent episode of diarrhoea. Diarrhoea or respiratory symptoms are often accompanied with fever, making it difficult for respondents to differentiate the associated symptoms. So, when fever was reported with either diarrhoea or acute respiratory infection, we only administered the detailed questionnaire relating to symptoms of diarrhoea or acute respiratory infection. If both diarrhoea and pneumonia were reported, questions asked were relevant to both the syndromes.

### Analysis of data

Data were entered from each healthcare-use survey form into separate Microsoft ACCESS 2003 database tables. Cleaning, management, and analysis of data were performed in the SAS for Windows software (version 8.0). Exact confidence intervals around proportions for prevalence of diseases and healthcare-use were calculated using the inverse Fisher F probability function in the Microsoft EXCEL 2003 software (Microsoft Corporation, Redmond, WA). The χ^2^ test was used for differences in proportions (SAS Institute, Cary, NC).

### Ethical approval

The Ethical Review Committee of KEMRI and the Investigational Review Board of CDC, Atlanta, reviewed and approved the protocol for conducting the HUS. Informed consent to participate in the HUS was obtained from all household caretakers or heads of households with under-five children.

## RESULTS

### Census

Census results indicated that there were 29,015 residents in 8,976 households in Gatwikira and Soweto villages (average household-size=3.2 individuals per household). While the Luo tribe comprised the majority (60%) of people within the study population at the time of the census, multiple other tribes were represented. The median age was 20 years. Fifteen percent of the population comprised under-five children, and <10% were aged over 40 years (Table 1). Most (88%) adults (n=11,292) within the study area indicated that they have spent at least 10 months per year within the Kibera settlement.

**Table 1.** Age distribution of residents in Gatwikira and Soweto villages, July 2005*

Age-category (years)	No.	%
0-1	1,130	4.4
2-4	2,703	10.6
5-19	8,422	33.0
20-29	7,185	28.1
30-39	3,850	15.1
40-49	1,605	6.3
50-59	508	2.0
60+	131	0.5
Total	25,534	100

*Data missing for 12% of the population

Females aged over 18 years were more likely not to have received any schooling compared to males in the same age-category [22% (95% confidence interval (CI) 20.9-23.2) vs 12% (95% CI 11.3-12.8)] (Table 2). Males aged over 18 years were more likely to have completed secondary schooling [30% (95% CI 28.6-30.6)] than females [17% (95% CI 16.5-18.6)] (Table 2). There were also considerable gender differences in employment status; 61% (95% CI 59.4-62.2) of females aged >18 years were unemployed compared to 9% (95% CI 8.8-10.0) of males. The census asked no questions about rent or income; so, source of light was used as a proxy for socioeconomic status. Only 15% (95% CI 14.8-16.3) of the households used electricity as their primary lighting source; the most prevalent lighting source was a tin-lamp [55% (95% CI 54.1-56.2)].

**Table 2.** Highest level of education completed by Gatwikira and Soweto residents aged over 18 years, July 2005*

Education	Males			Females		
No.	%	95% CI	No.	%	95% CI
Primary	4,194	53.3	52.2-54.4	2,927	57.3	56.0-58.7
Post-primary	163	2.1	1.8-2.4	62	1.2	1.0-1.6
Secondary	2,330	29.6	28.6-30.6	896	17.5	16.5-18.6
College	187	2.4	2.1-2.7	83	1.6	1.3-2.0
University	48	0.6	0.5-0.8	11	0.2	0.1-0.4
None	947	12.0	11.3-12.8	1,127	22.1	20.9-23.2
Total	7,869	100		5,106	100	

*Data missing for 521 males and 202 females.

CI=Confidence interval

### Healthcare-use study

#### Demographics and household characteristics

In total, 1,542 households (17% of total households and 54% of identified households) with under-five children were included in the HUS. Households with under-five children which were not included were those where no one was present at home during at least one visit by the field staff. The typical (90%) participating family lived in a one-room structure with rental fees of Ksh 300-500 per month (US$ 4.50-7.00 in 2005), with an average household-size of 5.1 (range 1-15) members. The head of the household for most households was a male (90%); 96% had a female caretaker residing within the household. Primary schooling was the most frequent highest level of education completed by the caretakers (58%) and the heads of households (45%). Secondary schooling was completed by 11% of the caretakers and 29% of the heads of households. In addition, 29% of the caretakers and 63% of the heads of households had completed a vocational training.

The majority (59%) of the family caretakers were unemployed while 26% were self-employed at least five days per week. Heads of the households were typically (95%) employed, with 52% having a regular salary, and the remaining self-employed, a category which included casual (daily) labourers; these figures are different from the numbers reported (above) from the census, which included residents without under-five children in the household. The majority (63%) of the caretakers indicated that they were always with the children while 23% were away for at least two hours during the day. More than half (51%) of the caretakers stayed in Kibera for at least 11 months of the year; 39% were away from Kibera for 1-5 months, and 9% were away for more than six months.

### Prevalence of syndromes

The syndrome-prevalence estimates were based upon a total of 9,110 residents, 6,290 (69%) of whom were aged ≥5 years, and 2,820 (31%) were aged less than five years. Overall, 1,378 (49%) of the under-five children and 1,139 (18%) of children and adults aged ≥5 years had been ill with at least one of the syndromes asked about. There were 466 (5.4%) hospitalizations during the two weeks before interview, 201 (43%) of which were for under-five children.

The prevalence of each of the syndromes was higher among the under-five children when compared with persons aged five years and above (Table 3). Some syndromes were substantially different; for instance, fever within the last two weeks was reported among 33% of the under-five children (95% CI 31.4-35.0) compared to 10% (95% CI 9.3-10.7) of people aged ≥5 years, and diarrhoea within the previous two weeks was reported in 23% (95% CI 21.3-24.4) of the under-five children and in 5% (95% CI 4.5-5.6%) of the older children and adults.

**Table 3.** Distribution of reported illness syndromes among healthcare-use survey respondents by age-category, Gatwikera and Soweto villages, Kibera, 2005

Age-category (years)	No. of survey respondents*	% reported pneumonia in the past year (95% CI)	% reported ARI in the past 2 weeks (95% CI)	% reported diarrhoea in the past 2 weeks (95% CI)	% reported fever in the past 2 weeks (95% CI)
0-4	2,778	8.9 (7.9-10.1)	12.0 (10.8-13.3)	22.8 (21.3-24.4)	33.2 (31.4-35.0)
5-17	2,331	5.1 (4.3-6.1)	6.0 (5.0-7.0)	6.1 (5.2-7.2)	10.7 (9.5-12.1)
18-49	3,937	3.9 (3.3-4.5)	5.7 (5.0-6.5)	4.4 (3.8-5.1)	9.7 (8.8-10.6)
>50	65	3.0 (0.4-10.4)	4.5 (0.9-12.5)	4.4 (0.9-12.4)	11.6 (4.1-21.6)
All ages	8,580	5.7 (5.2-6.2)	7.7 (7.2-8.3)	10.5 (9.9-11.1)	17.0 (16.3-17.8)

*The highest number of respondents per age-category. There were slight variations in response rates depending upon illness syndrome.

ARI=Acute respiratory illness

CI=Confidence interval

### Healthcare-use

For participants with a history of diarrhoeal disease, some sort of healthcare outside the home was sought for 368 (82%) under-five children (Table 4) and for 148 (77%) of older children and adults during the previous two weeks. Of 470 healthcare encounters for under-five children with diarrhoeal disease (some had >1 healthcare encounter), 57% were at a clinic or a hospital, 38.7% were with a chemist or a kiosk selling medicines, and 1.5% were reported to be with a spiritual healer (the remaining 2% of the encounters were with unlicensed healthcare providers). Of 65 under-five children with a diarrhoeal illness associated with >1 healthcare encounter, the most common first encounter was with a chemist or a kiosk in 30 (46%). When combined with data from patients with only one visit, 198 (54%) first visits were at a licensed clinic or a hospital, 160 (43%) of the first (or only) visits were with a chemist or a kiosk, and 10 (2.7%) were with a spiritual healer or an unlicensed healthcare provider.

**Table 4.** Healthcare-seeking behaviours for under-five children, by reported illness, Gatwikera and Soweto villages, Kibera, Kenya, 2005

Healthcare-seeking behaviour	% of children with reported pneumonia in the past year (95% CI) (n=153)	% of children with reported ARI in the past 2 weeks (95% CI) (n=292)	% of children with reported diarrhoea in the past 2 weeks (95% CI) (n=448)	% of children with reported fever in the past 2 weeks (95% CI) (n=384)
Healthcare-seeking behaviours outside household				
Any care sought outside household	98.7 (95.4-99.8)	87.3 (83.0-90.9)	81.7 (77.8-85.2)	85.2 (81.2-88.6)
Visited any healthcare facility	92.8 (87.5-96.4)	34.2 (28.8-40.0)	52.5 (47.7-57.2)	54.7 (49.6-59.7)
Visited a hospital	51.5 (42.7-60.2)	26.0 (20.3-32.4)	17.4 (13.4-22.0)	11.4 (8.0-15.6)
Hospitalization	30.6 (22.9-39.1)	13.2 (9.1-18.5)	6.8 (4.3-10.2)	2.7 (1.2-5.2)
Private care providers	6.7 (3.1-12.4)	4.1 (1.9-7.7)	3.7 (1.9-6.4)	5.7 (3.4-9.0)
Drug-sellers	27.6 (20.2-36.0)	45.7 (38.9-52.5)	54.3 (48.7-59.9)	49.7 (43.8-55.5)
Traditional healers	6.0 (2.6-11.4)	0.9 (0.1-3.3)	3.1 (1.5-5.6)	1.7 (0.5-3.9)
Village health volunteers	0.0 (0.0-2.2)	0.5 (0.0-2.5)	0.0 (0.0-0.9)	1.0 (0.2-2.9)
Reported diagnostics				
Chest radiograph	19.4 (13.1-27.1)	8.2 (4.9-12.7)	NA*	NA*
Blood smear	59.7 (50.9-68.1)	36.5 (30.1-43.3)	26.7 (22.0-31.9)	25.2 (20.3-30.5)
Reported treatments				
Antibiotics	91.8 (85.8-95.8)	81.7 (76.0-86.6)	77.6 (72.7-82.1)	61.1 (55.3-66.6)
Antimalarial medication	59.0 (50.1-67.4)	55.3 (48.4-62.0)	41.3 (35.9-46.9)	45.0 (39.2-50.8)
Oral rehydration therapy	38.8 (30.5-47.6)	34.2 (28.0-40.9)	48.8 (43.2-54.4)	16.4 (12.4-21.1)
Fever-reducer/pain killer	88.1 (81.3-93.0)	75.3 (69.1-80.9)	71.1 (65.8-76.0)	80.2 (75.2-84.6)

*Only responded if illness was pneumonia or ARI.

ARI=Acute respiratory illness

CI=Confidence interval

NA=Not applicable

For ARI episodes occurring within the previous two weeks, 255 (87%) of the under-five children (Table 4) and 204 (81%) of the older children and adults sought some healthcare outside the home. Of 333 encounters in under-five children for acute respiratory infection, 67% were at a clinic or a hospital, 30% were with a chemist or a kiosk, and 1.8% were with a spiritual healer. Of the 234 first (or only) visits, the most common care provider (150 visits, 64%) was at a licensed clinic or a hospital, 78 (34%) were with a chemist or a kiosk, and five (2.1%) were with a spiritual healer or an unlicensed healthcare provider.

For 316 health encounters for under-five children with pneumonia during the past year, a licensed clinic or a hospital was overwhelmingly the most prevalent option representing 251 (79%) visits, followed by chemists or kiosks (46 visits, 15%) and spiritual healers/unlicensed healthcare providers (19 visits, 6.0%). Since pneumonia is a technical term often used by a physician or other licensed healthcare providers at clinics or hospitals, questions about healthcare-use for a technical diagnosis such as pneumonia likely biased towards having a clinic or hospital visit associated with that illness; so, we may not have optimally measured healthcare-use for pneumonia.

For febrile episodes occurring within the previous two weeks, healthcare outside the home was sought for 327 (85%) under-five children (Table 4) and for 246 (84%) older children and adults. Of 419 healthcare encounters for fever in under-five children, 57% were at a clinic or a hospital, 36% were with a chemist or a kiosk, and 3.8% were with a spiritual healer. Of the 335 first visits, 185 (55%) were at a licensed clinic or a hospital. The next most common first (or only) visit was with a chemist or a kiosk (132 visits, 39%), and 18 (5.4%) were with a spiritual healer or an unlicensed healthcare provider.

All healthcare-use for the under-five children was significantly lower [83% (95% CI 80.6-86.2)] in households where caretakers spent all their time at home with children than where caretakers spent an average of 5-7 hours per day away from children [(93% (95% CI 86.3-97.6; p=0.01 for the comparison)] or spent any time away from children [90% (95% CI 86.5-92.2; p<0.01 for the comparison)] (Table 5). The higher the educational level of the head of the household, the greater was the tendency for members of the household to seek care for illness, although this relationship was not significant (p=0.18) (Table 5)]. However, the employment status of the caretaker was not associated with healthcare-use for under-five children [89% (95% CI 78.1-95.3) for unemployed vs 86% (95% CI 83.7-87.8) for employed] (p=0.53). There were also no significant differences in healthcare-use depending upon time spent away from Kibera. Finally, there were no differences in healthcare-seeking behaviour when stratified by reported monthly expenditure on rent (Table 5).

**Table 5.** Analysis of background factors and any type of healthcare-use

Background factor	Less than 5 years of age					5 years of age and older				
	Sought healthcare?			Point estimate (%) (95% CI)		Sought healthcare?			Point estimate (%) (95% CI)	
	Yes	No	Total			Yes	No	Total		
Highest education of caretaker										
No schooling	34	4	38	89.5 (75.2-97.1)		26	4	30	86.7 (69.3-96.2)	
<Primary	271	63	334	81.1 (76.5-85.2)		184	42	226	81.4 (75.7-86.3)	
Primary	610	91	701	87.0 (84.3-89.4)		414	79	493	84.0 (80.4-87.1)	
Secondary	111	10	121	91.7 (85.3-96.0)		70	10	80	87.5 (78.2-93.8)	
Diploma	7	1	8	87.5 (47.3-99.7)						
Sex of caretaker										
Male	31	2	33	93.9 (79.8-99.3)		31	0	31	100 (90.8-100.0)	
Female	1,004	167	1,171	85.7 (83.6-87.7)		664	136	800	83.0 (80.2-85.5)	
Sex of head of household										
Male	926	147	1,073	86.3 (84.1-88.3)		622	117	739	84.2 (81.3-86.7)	
Female	105	21	126	83.3 (75.7-89.4)		68	19	87	78.2 (68.0-86.3)	
Vocational training of caretaker										
No	286	39	325	88.0 (84.0-91.3)		212	30	242	87.6 (82.8-91.5)	
Yes	739	127	866	85.3 (82.8-87.6)		474	105	579	81.9 (78.5-84.9)	
Highest education of head of household										
No schooling	9	1	10	90.0 (55.5-99.7)		3	1	4	75.0 (19.4-99.4)	
<Primary	106	22	128	82.8 (75.1-88.9)		54	11	65	83.1 (71.7-91.2)	
Primary	471	84	555	84.9 (81.6-87.7)		310	57	367	84.5 (80.4-88.0)	
Secondary	288	45	333	86.5 (82.3-90.0)		198	47	245	80.8 (75.3-85.6)	
Diploma	12	1	13	92.3 (64.0-99.8)		13	1	14	92.9 (66.1-99.8)	
Bachelor's	4	0	4	100.0 (47.3-100,0)		3	1	4	75.0 (19.4-99.4)	
>Bachelor's	3	0	3	100.0 (36.8-100.0)		4	0	4	100 (47.3-100.0)	
Vocational training of head of household										
No	592	94	686	86.3 (83.5-88.8)		394	67	461	85.5 (81.9-88.6)	
Yes	338	63	401	84.3 (80.4-87.7)		225	57	282	79.8 (74.6-84.3)	
Head of household self-employed GE 5 days										
No	829	136	965	85.9 (83.6-88.0)		550	118	668	82.3 (79.2-85.2)	
Yes	200	32	232	86.2 (81.1-90.4)		147	18	165	89.1 (83.3-93.4)	
Background factor	Less than 5 years of age					Five years of age and older				
	Sought healthcare?			Point estimate (%) (95% CI)		Sought healthcare?			Point estimate (%) (95% CI)	
	Yes	No	Total			Yes	No	Total		
Head of household self-employed <5 days										
No	931	157	1,088	85.6 (83.3-87.6)		628	121	749	83.8 (81.0-86.4)	
Yes	101	11	112	90.2 (83.1-95.0)		70	15	85	82.4 (72.6-89.8)	
Head of household salaried GE 5 days/week										
No	986	163	1,149	85.8 (83.7-87.8)		661	163	824	80.2 (77.3-82.9)	
Yes	46	5	51	90.2 (78.6-96.7)		46	5	51	90.2 (78.6-96.7)	
Head of household salaried <5 days/week										
No	1,004	166	1,170	85.8 (83.7-87.8)		679	126	805	84.3 (81.7-86.8)	
Yes	28	2	30	93.3 (77.9-99.2)		19	10	29	65.5 (45.7-82.1)	
Head of household unemployed										
No	370	46	416	88.9 (85.5-91.8)		274	43	317	86.4 (82.2-90.0)	
Yes	661	121	782	84.5 (81.8-87.0)		422	93	515	81.9 (78.3-85.2)	
Caretaker self-employed **≥**5 days										
No	668	104	772	86.5	(83.9-88.9)	490	94	584	83.9 (80.7-86.8)	
Yes	358	63	421	85.0	(81.3-88.3)	204	41	245	83.3 (78.0-87.7)	
Caretaker self-employed <5 days										
No	918	156	1,074	85.5 (83.2-87.5)		620	115	735	84.4 (81.5-86.9)	
Yes	109	11	120	90.8 (84.2-95.3)		74	19	93	79.6 (69.9-87.2)	
Caretaker salaried GE 5 days/week										
No	713	112	825	86.4 (83.9-88.7)		466	98	564	82.6 (79.2-85.7)	
Yes	316	55	371	85.2 (81.1-88.6)		230	37	267	86.1 (81.4-90.1)	
Caretaker salaried <5 days/week										
No	933	141	1,074	86.9 (84.7-88.8)		631	120	751	84.0 (81.2-86.6)	
Yes	96	26	122	78.7 (70.4-85.6)		65	15	80	81.3 (71.0-89.1)	
Caretaker unemployed										
No	972	160	1,132	85.9 (83.7-87.8)		662	124	786	84.2 (81.5-86.7)	
Yes	55	7	62	88.7 (78.1-95.3)		33	11	44	75.0 (59.7-86.8)	
Background factor	Less than 5 years of age					Five years of age and older				
	Sought healthcare?			Point estimate (%) (95% CI)		Sought healthcare?			Point estimate (%) (95% CI)	
	Yes	No	Total			Yes	No	Total		
Time caretaker spends away from kids										
Always at home	609	120	729	83.5 (80.6-86.2)		402	87	489	82.2 (78.5-85.5)	
<1 hour	103	13	116	88.8 (81.6-93.9)		57	12	69	82.6 (71.6-90.7)	
2-4 hours	162	22	184	88.0 (82.5-92.4)		108	17	125	86.4 (79.1-91.9)	
5-7 hours	86	6	92	93.5 (86.3-97.6)		81	9	90	90.0	(81.9-95.3)
8-10 hours	58	7	65	89.2 (79.1-95.6)		39	9	48	81.3 (67.4-91.1)	
>10 hours	12	1	13	92.3 (64.0-99.8)		11	2	13	84.6 (54.6-98.1)	
Anytime away from home	421	49	470	89.6 (86.5-92.2)		296	49	345	85.8 (81.7-89.3)	
Time (months) caretaker spends up country										
Always in Kibera	158	34	192	82.3 (76.1-87.4)		116	26	142	81.7 (74.3-87.7)	
<1	335	47	382	87.7 (84.0-90.8)		272	51	323	84.2 (79.8-88.0)	
1-5	428	68	496	86.3 (82.9-89.2)		248	47	295	84.1 (79.4-88.1)	
6-9	64	11	75	85.3 (75.3-92.4)		32	7	39	82.1 (66.5-92.5)	
>10	44	8	52	84.6 (71.9-93.1)		21	5	26	80.8 (60.6-93.4)	
Rent/month (Ksh)										
None, owns home	17	0	17	100 (83.8-100.0)		16	2	18	88.9 (65.3-98.6)	
None, does not pay rent	3	0	3	100 (36.8-100.0)		1	2	3	33.3 (0.8-90.6)	
<200	3	0	3	100 (36.8-100.0)		6	0	6	100 (60.7-100.0)	
300-500	599	96	695	86.2 (83.4-88.7)		374	79	453	82.6 (78.7-85.9)	
600-800	275	49	324	84.9 (80.5-88.6)		185	33	218	84.9 (79.4-89.3)	
900-1,200	96	15	111	86.5 (78.7-92.2)		76	14	90	84.4 (75.3-91.2)	
>12,000	38	6	44	86.4 (72.6-94.8)		35	4	39	89.7 (75.8-97.1)	

Although some form of healthcare was sought in most (85%) cases for all the syndromes surveyed, 324 patients across the syndromes did not seek healthcare; 162 (50%) of them indicated that healthcare was very expensive, when asked why healthcare was not sought despite illness (among various potential responses provided to respondents). Other leading reasons cited for not using healthcare included the child/adult ‘not being sick enough’ (27%), and the sick individual was ‘getting better on their own’ (13%). Distance to a healthcare facility was only cited as a barrier in 1.6% of the cases. In 24% of the cases, when the hospital or clinic was not the primary source of healthcare, it had been advised by a healthcare provider that the patient should visit the hospital; 44% of the time these individuals did visit the hospital. The most common reason cited for not proceeding to the hospital after being advised to do so was cost (27%).

Healthcare-use was not significantly different by sex of the patient when stratified by syndrome and age-category, except that males aged ≥5 years sought healthcare for episodes of fever [93% (95% CI 85.9-96.8)] compared to 80% of females aged ≥5 years (95% CI 73.5-85.7) (p<0.005). When children aged 5-16 years were compared with adults aged >17 years, there were no significant differences in the healthcare-use patterns, except when considering pneumonia for which children were more likely to visit a healthcare facility than adults (88% vs 67%; p<0.01), and for diarrhoea, children were more likely than adults to receive oral rehydration therapy (57% vs 23%; p<0.0001).

## DISCUSSION

Massive urbanization has led to crowded residential communities with poor sanitation, water and air quality. This has resulted in various stresses on health systems with potential implications on disease-incidence rates and rapid spread of recognized and emerging infectious diseases. Because of this potential, we have established a population-based surveillance for key infectious disease syndromes and their aetiologies in Kibera, a large urban slum in East Africa. This healthcare-use study has provided baseline data for this surveillance programme of infectious disease syndromes.

The findings of the study suggest that a high proportion of illnesses in this urban setting is associated with some form of healthcare-seeking, often including informal kiosks or unlicensed care providers. However, a substantial number of patients, regardless of age, living within informal settlements do not appear to use the multiple licensed healthcare-delivery options available within urban environments. This is consistent with the recent findings of another study of informal settlements in Nairobi (9). However, the proportion of patients accessing healthcare facilities in our study was higher for diarrhoeal disease and for fever (but not for respiratory diseases in under-five children) than that reported from a rural area in Kenya during a healthcare-use study conducted around the same time (10).

Healthcare-use was not significantly different among the two age-categories (under-five children and older children and adults aged ≥5 years), although under-five children are often at special risk of serious outcomes from illness. The results are not in agreement with that from another slum setting in Nairobi, which showed that infants were prioritized by caretakers for healthcare when compared with older children (11). Despite being an impoverished population, most people sought some sort of healthcare for illness outside the home. Although the majority of the patients visited licensed clinics and hospitals, >35% of the patients with respiratory, febrile or diarrhoeal illness only visited chemists (pharmacists) and unlicensed kiosks selling drugs or other unlicensed care providers instead of licensed clinics. Reasons given for not seeking healthcare suggest inability to meet costs, along with inconvenience. These findings have important implications for the delivery of healthcare and for public-health interventions. These also suggest that comprehensive surveillance for infectious disease syndromes and emerging pathogens in an urban setting would either need to include a large number of clinics, hospitals, and kiosks and informal care providers, or would require community-based approaches, or ideally, a combination of the two.

While most (85%) people sought some sort of healthcare, the most common reason reported for not seeking healthcare was cost. The Kenya National Health Accounts for 2002 (published in 2005) indicated that households paid for 51% of all healthcare costs, and per-capita annual health costs were about Ksh 1,500 (US$ 19 in 2002) (12). Given that >55% of the Kenyan population is classified as ‘poor’, cost likely represents a substantial barrier to appropriate levels of healthcare. In fact, the same survey found that about one-third of ill people classified as ‘poor’ did not seek healthcare compared to 15% of the better-off (12). The implications for our surveillance activity are as follows: to optimize detection of illnesses (so that the disease can be characterized and specimens collected and tested), health services would need to be provided without consideration of cost to the population under surveillance.

The span of time a caretaker spends in the home with children was associated with lower healthcare-use for under-five household members, especially when the caretaker is always at home. We cannot confidently explain this finding. Perhaps, such caretakers are not being able to seek healthcare outside the household for one child because they cannot leave the house due to other children who must be cared for. However, not having anyone available to take care of other children was only cited as a reason for not seeking healthcare in less than 1% of the time. Another possibility is that women who were always at home were less likely to have income and to have available funds to pay for care. Alternatively, caretakers who are always at home may be serving as a healthcare-use ‘proxy’, able to monitor the child's status in the home, and only seeking healthcare when the condition worsens.

Over the next two decades, the urban population within the developing world is expected to grow by nearly three billion while the rural population is projected to increase by nearly 500 million (1). Urban migration has been particularly pronounced in Africa (1) where perceived opportunities for generation of income and education have encouraged mobility at the expense of key factors, such as sanitation, hygiene, and dense population, which can impact the quality of life. Urban centres often do not have the infrastructure to handle the influx of people spawning informal settlements which have expanded rapidly in many cities in Africa. From 1971 through 1995, the share of Nairobi residents residing within informal settlements increased from roughly one-third to >60%, representing a population increase from 167,000 to nearly two million (13).

The findings of this survey suggest that diarrhoeal, respiratory and febrile illnesses occur commonly in Kibera and that while under-five children appear to be at particularly high risk of illness, there is also substantial morbidity among older children and adults. We cannot characterize the incidence rates of these syndromes based on this survey since its timing (mid-July) does not allow seasonality to be taken into account. Active surveillance which began in 2006 will provide more reliable data on the incidence of key infectious disease syndromes and their aetiologies and the ability to characterize the risk factors and transmission patterns for specific pathogens. The findings of this study suggest the need for a community rather than facility-based design to achieve our objectives for systematic disease surveillance in this setting, given the high proportion of illnesses not associated with visit to a healthcare facility (and a large number of various types of healthcare facilities available within this urban environment)

The ‘self-reporting’ nature of the study likely led to misclassifications of illness, especially for pneumonia and fever. For example, while the assessment of diarrhoea is fairly straightforward, the assessment of fever is more subjective, especially when thermometers are rarely used. ARI is even more subjective as the case definition of ‘cough and difficult breathing’ does not necessarily exclude upper respiratory tract infections or bronchitis. Such non-specific measures have the potential to bias estimates of prevalence upward and potentially blur the relationships between valid syndromes and associated appropriate healthcare-use.

As noted above, the findings of this study conducted within an urban informal settlement differed from a similar study in 2005 that we conducted within a rural Kenyan area (10). Within the rural setting, distance of residence to healthcare facilities played a much larger role in healthcare-seeking behaviour. While the proportion of patients accessing clinics and hospitals for diarrhoeal disease and fever was higher in the urban setting, both studies confirmed that facility-based disease surveillance (without other components to access patient information and relevant specimens) would substantially underestimate the burden of diseases due to specific aetiologies. In addition, the proportions of the respondents and children reporting diarrhoeal and respiratory diseases (but not febrile illness) were higher within the urban area, possibly suggesting an impact of densely-populated environments with sub-optimal sanitation but also potentially influenced by different tendencies to recognize and/or report an illness in urban versus rural areas. Confirmation and characterization of differences and similarities will await results of active, comprehensive, community-based surveillance now ongoing within villages in Kibera and in rural western Kenya. In addition to implications for surveillance methodologies and interpretation of surveillance data, the findings of the present study suggest that strategies are needed to ensure that the massively-increasing number of urban residents has optimal access to medical care and treatment.

## ACKNOWLEDGEMENTS

The authors are grateful for the work of the community interviewers and other KEMRI-CDC staff who work in Kibera, the administrative support of Evelyne Mulama, and the continuing cooperation and support from the people of Gatwikera and west Soweto in Kibera. This paper is approved with the permission of the Director KEMRI.

## References

[1] Population Reference Bureau (2008). 2008 world population data sheet.

[2] United Nations Human Settlements Programme (2010). The state of African cities 2010: governance, inequality and urban land markets.

[3] Arnaud A (1998). The challenges of urbanization.

[4] Venard JL (1995). Urban planning and environment in sub-Saharan Africa. Building blocks for Africa 2025..

[5] Patel RB, Burke TF (2009). Urbanization—an emerging humanitarian disaster. N Engl J Med.

[6] Sikolia DN, Mwololo K, Cherop H, Hussein A, Juma M, Kurui J (2002). The prevalence of acute respiratory infections and the associated risk factors: a study of children under five years of age in Kibera Lindi village, Nairobi, Kenya. J Nat Inst Public Health.

[7] Gulis G, Mulumba JAA, Juma O, Kakosova B (2004). Health status of people of slums in Nairobi, Kenya. Environ Res.

[8] Develay A, Sauerborn R, Diesfeld HJ (1996). Utilization of health care in an African urban area: results from a household survey in Ouagadougou, Burkina-Faso. Soc Sci Med.

[9] Ndugwa RP, Zulu EM (2008). Child morbidity and care-seeking in Nairobi slum settlements: the role of environmental and socio-economic factors. J Child Health Care.

[10] Burton D, Flannery B, Onyango B, Larson C, Alaii J, Zhang X (2011). Health-seeking behavior for common infectious disease illnesses in rural Kenya. J Health Popul Nutr.

[11] Taffa N, Chepngeno G, Amuyunzu-Nyamongo M (2005). Child morbidity and healthcare utilization in the slums of Nairobi, Kenya. J Trop Pediatr.

[12] Kenya (2005). Ministry of Health. National health accounts—NHA country policy briefs. Kenya National Health Accounts (NHA) 2002: estimating expenditures on general health and HIV/AIDS care.

[13] United Nations Human Settlements Programme (2003). The challenges of slums: global report on human settlements 2003.

